# NNZ-2566 treatment inhibits neuroinflammation and pro-inflammatory cytokine expression induced by experimental penetrating ballistic-like brain injury in rats

**DOI:** 10.1186/1742-2094-6-19

**Published:** 2009-08-05

**Authors:** Hans H Wei, Xi-Chun M Lu, Deborah A Shear, Anu Waghray, Changping Yao, Frank C Tortella, Jitendra R Dave

**Affiliations:** 1 Department of Applied Neurobiology, Division of Psychiatry and Neuroscience, Walter Reed Army Institute of Research, Silver Spring, Maryland 20910, USA

## Abstract

**Background:**

Inflammatory cytokines play a crucial role in the pathophysiology of traumatic brain injury (TBI), exerting either deleterious effects on the progression of tissue damage or beneficial roles during recovery and repair. NNZ-2566, a synthetic analogue of the neuroprotective tripeptide Glypromate^®^, has been shown to be neuroprotective in animal models of brain injury. The goal of this study was to determine the effects of NNZ-2566 on inflammatory cytokine expression and neuroinflammation induced by penetrating ballistic-like brain injury (PBBI) in rats.

**Methods:**

NNZ-2566 or vehicle (saline) was administered intravenously as a bolus injection (10 mg/kg) at 30 min post-injury, immediately followed by a continuous infusion of NNZ-2566 (3 mg/kg/h), or equal volume of vehicle, for various durations. Inflammatory cytokine gene expression from the brain tissue of rats exposed to PBBI was evaluated using microarray, quantitative real time PCR (QRT-PCR), and enzyme-linked immunosorbent assay (ELISA) array. Histopathology of the injured brains was examined using hematoxylin and eosin (H&E) and immunocytochemistry of inflammatory cytokine IL-1β.

**Results:**

NNZ-2566 treatment significantly reduced injury-mediated up-regulation of IL-1β, TNF-α, E-selectin and IL-6 mRNA during the acute injury phase. ELISA cytokine array showed that NZ-2566 treatment significantly reduced levels of the pro-inflammatory cytokines IL-1β, TNF-α and IFN-γ in the injured brain, but did not affect anti-inflammatory cytokine IL-6 levels.

**Conclusion:**

Collectively, these results suggest that the neuroprotective effects of NNZ-2566 may, in part, be functionally attributed to the compound's ability to modulate expression of multiple neuroinflammatory mediators in the injured brain.

## Background

In the United States, traumatic brain injury (TBI) is the primary cause of death and disability in persons under 45 years old, occurring more frequently than breast cancer, HIV-AIDS, multiple sclerosis, and spinal cord injury combined [1,2]. Overall, the leading causes of TBI are falls and motor vehicle accidents; however, penetrating ballistic-like brain injury (PBBI) represents one of the most severe TBI categories and is the leading cause of TBI-related death in both civilian and military populations [3,4].

Experimental studies of PBBI have demonstrated a rapid activation and recruitment of inflammatory resident glial cells, astrocytes, microglia, and blood leukocytes accumulated in the injured brain that secretes soluble pro-inflammatory cytokines [5-7]. Although cerebral inflammation can play both a beneficial and a detrimental role in the injury repair process [8,9], reactive glial cells and leukocytes secrete a variety of neurotoxic molecules which likely contribute to progressive neuronal death after TBI [9-11]. Treatment strategies targeting the more acute inflammatory events in hypoxic-ischemic injury and other TBI models have demonstrated that a reduction in leukocyte infiltration into the injured brain can improve both histopathological and functional outcomes [12,13].

NNZ-2566 is a synthetic analogue of the endogenous N-terminus tripeptide, Glycine-Proline-Glutamate (GPE, Glypromate^®^; Neuren Pharmaceuticals), which is proteolytically cleaved from insulin-like growth factor-1 (IGF-1) in the brain [14-19]. GPE has been shown to cross the blood-brain barrier and protect against cell death both in vitro [20,21] and in vivo [22,23] but is rapidly metabolized [22,23]. GPE has been shown to have potent neuroprotective effects in animal models of hypoxic-ischemic brain injury [23,24] and neurodegenerative disease [20]. The GPE analogue, NNZ-2566, was designed to have an extended (> 70 minute) half-life in order to optimize its therapeutic potential. Most recently, the results of a comprehensive, dose-response study demonstrated that treatment with NNZ-2566 protects against PBBI-induced inflammation and apoptosis and promotes functional recovery [25]. The present study was designed to further elucidate mechanisms of NNZ-2566-mediated neuroprotection by assessing its effect on PBBI-induced up-regulation of pro-inflammatory cytokines in both the acute (4 h-3 day-) and chronic (7 day) post-injury periods.

## Methods

### Design

Male Sprague-Dawley rats (250–300 g; Charles River Labs, Raleigh, VA) were used for this study, and all procedures were approved by the Walter Reed Army Institute of Research Animal Care and Use Committee. Research was conducted in compliance with the Animal Welfare Act and other federal statutes and regulations relating to animals and experiments involving animals and adhered to principles stated in the Guide for the Care and Use of Laboratory Animals (NRC Publication, 1996 edition). Animals were housed individually under a 12 h light/dark cycle in a facility accredited by the Association for Assessment and Accreditation of Laboratory Animal Care International.

#### Penetrating ballistic brain injury and treatments

The Dragonfly Model # HPD-1700 Variable Pressure Waveform Generator and PBBI probe (Dragonfly Inc., WV) were used to simulate a right, frontal ballistic injury to the rat brain [5]. Rats were anesthetized using 2% isoflurane delivered in oxygen and positioned in a stereotaxic frame for probe insertion (Kopf, Tujunga, CA). Normothermia (37 ± 1°C) was maintained throughout the surgical procedure by means of a homoeothermic heating system (Harvard Apparatus, South Natick, MA). PBBI was induced by delivery of a pressure pulse calibrated to rapidly inflate/deflate the PBBI balloon to a diameter of 0.633 cm, which is 10% of total rat brain volume. A unilateral frontal hemispheric injury at a 10% severity level represents a survivable injury associated with well-defined, consistent and reproducible histopathological damage [5,26]. After surgery, animals were placed in a warm heating blanket until they recovered from anesthesia and food and water were provided *ad libitum*. Sham animals were not subjected to probe insertion but otherwise received all surgical manipulations including anesthesia, scalp incision, and craniotomy.

Three groups of eight rats were evaluated: vehicle/sham, vehicle/PBBI, NNZ-2566/PBBI. A bolus injection of 10 mg/kg NNZ-2566 or 1 ml/kg saline (vehicle) was administered intravenously (IV) to each animal at 30 minutes post-PBBI surgery, immediately followed by a continuous IV infusion of NNZ-2566 at a rate of 3 mg/kg/h or an equal volume of vehicle for various durations (1 h, 4 h, or 12 h). Rats were subsequently euthanized and brain tissues collected for processing at 1 h, 4 h, 12 h, 24 h, 3, and 7 days following the initiation of treatment.

#### Histopathology

At 3 and 7 days post-PBBI, animals were anesthetized as described above and transcardially perfused with phosphate buffered saline (PBS, pH 7.4 at room temperature) followed by 4% paraformaldehyde in 4°C. Brains were extracted, immersed in 4% paraformaldehyde for 6 h and then transferred to 0.1 M phosphate buffer containing 20% sucrose (pH 7.4, 4°C). All brain tissues were sent to FD Neurotechnologies (Baltimore, MD) for histopathological and immunohistochemical processing where coronal brain sections (40 μm thick) were cut through the cerebrum from +4.0 to -7.0 mm AP to bregma with serial sections collected at 480 μm intervals. Hematoxylin & Eosin (H&E) staining was used for morphological assessment of injury and detection of inflammatory cells including polymorphonucleocytes (neutrophils), monocytes, and macrophage-like cells defined by large irregular cytoplasm [27]. Brain sections were dehydrated in absolute ethanol. All sections were covered with cover slips in Permount^® ^(Fisher Scientific, Fair Lawn, NJ). Morphological changes in all tissue samples were visualized using light microscopy (20 × magnifications). Due to the limited number of samples available for each group (n = 2) these data were not quantified.

### Tissue processing for gene expression studies

At each endpoint, rats were deeply anesthetized with 70 mg/kg ketamine/6 mg/kg xylazine and fresh brain tissue was harvested for analysis. A 3-mm section was dissected from both ipsilateral and contralateral hemispheres of each rat brain (0–3 mm rostral to bregma), rapidly frozen on dry ice, and stored at -70°C for RNA extraction. Frozen brain tissue was homogenized in the lysis buffer, and total RNA was extracted using Qiagen RNeasy Liquid Tissue Mini Kit according to the manufacturer's instructions (Qiagen Science, Germantown, MD). RNA purity and concentration were determined spectrophotometrically by calculating the ratio between the absorbance at 260 nm and 280 nm. The absorbance ratio for all samples ranged from 1.8 to 2.1. The quality of RNA for all samples was confirmed by resolving on a 1.5% formaldehyde agarose gel.

### Microarray analysis

Microarray analysis of inflammatory cytokine gene expression was performed on mRNA using the Oligo GEArray Rat Inflammatory Cytokines and Receptors Microarray (Superarray Biosciences, Frederick, MD). The cDNA array membrane contained 112 inflammatory cytokines, chemokines, cytokine/chemokine receptors, and housekeeping genes. Ribosomal protein L32 (RPL32) and glyceraldehyde-3-phosphate dehydrogenase (GAPDH) genes were used as controls, and global changes in cytokine gene expression levels at 4 h following PBBI were compared in sham, vehicle and NNZ-2566 treated animals. Brain tissues from 6 animals in each group were pooled and samples were run in duplicate. Hybridization procedures were performed as per the manufacturer's instructions. Biotin-labeled cRNA probes were synthesized from total RNA using a TrueLabeling-AMP Linear RNA Amplification Kit (SuperArray Biosciences, Frederick, MD). The labeled cRNA probes were hybridized to oligonucleotide fragments spotted on the gene array membranes. Membranes were washed to remove any unincorporated probe and incubated with alkaline phosphatase-conjugated streptavidin (AP-streptavidin). The chemiluminescence array images, generated from the alkaline phosphatase substrate CDPStar, were captured by a Fuji LAS-3000 cooled CCD camera system. The images were analyzed using the web-based software GEArray Expression Analysis Suite (Superarray Biosciences, Fredercik, MD). The values of mRNA were normalized for the amount of RPL32 presented in each sample.

### Quantitative RT-PCR

Interleukin 1 beta (IL-1β), interleukin 6 (IL-6), tumor necrosis factor alpha (TNF-α) and E-selectin were selected for quantitative reverse transcriptase polymerase chain reaction (qRT-PCR) analysis to confirm the oligo-cDNA microarray findings. Reverse transcription reactions were carried out using RNA PCR Core Kit in a DNA Thermal Cycler 480 (Perkin-Elmer). PCR primers and TaqMan™ probes were designed using Primer Express 2.0 Software and synthesis was performed by *AB *Applied Biosystems. Primer/probe sequences were used according to the methods of Berti et al. [28]. Quantitative real time PCR was performed with ABI Prism 7000 sequence detection system (PE Applied Biosystems) as previously described [28] using TaqMan™ Universal PCR Master Mix (*AB *Applied Biosystems) as per the manufacturer's instructions. Amplification was achieved with thermal conditions of 2 minutes at 50°C and 10 minutes at 95°C. The samples were then run for 40 cycles each at 95°C for 15 seconds and 60°C for 1 minute. Data are presented as the relative induction of each cytokine or cellular adhesion molecule normalized to RPL32.

### Multiplex cytokine ELISA assay

Brain tissue was homogenized in the lysis buffer and centrifuged at 1500 *g *for 15 min at 4°C. The supernatant was collected and stored frozen at -70°C. Cytokines were quantified with samples containing 50 μg of protein using SearchLight Rat Cytokine Array (Pierce Endogen, Woburn, MA) according to the manufacturer's instructions.

### Immunostaining with the anti-IL-1β antibody

An additional series of brain sections were processed for immunostaining with anti-IL-1β antibody to detect PBBI-induced inflammatory cell and reactive gliosis response. The sections were incubated, free-floating, with IL-1β antibody (1:1000; R&D systems, AF-501-NA, 0.2 mg/ml) in 0.1 M PBS containing 1% normal horse serum and 0.3% Triton X-100 for 3 days at 4°C. The immunoreaction product was visualized according to the avidin-biotin complex method with Vectastin Elite ABC kit (Vector Laboratory, Burlingame, CA).

### Statistical analysis

Quantitative data (RT-PCR gene expression levels and cytokine levels in brain tissue) were analyzed using one way analysis of variance (ANOVA) followed by a Dunnett's post-hoc analysis for comparison between sham controls and treated groups at each time point. Data are presented as mean ± S.E.M. (*p *< 0.05 indicates significant difference among groups).

## Results

### Histopathology and inflammatory cell response (H&E staining)

**Figure 1:**
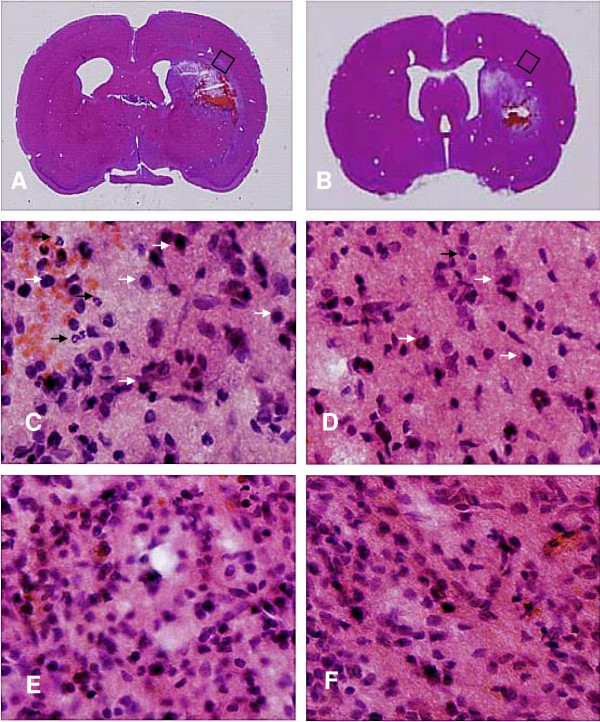
**NNZ-2566 treatment decreased inflammatory leukocyte filtration (H & E staining) after PBBI**. Neutrophils (black arrows) and large macrophage-like cells (white arrows) were prominent in area surrounding the lesion cavity 3 days following PBBI (A, C). NNZ-2566 treatment (B, D) inhibited the infiltration of neutrophils and macrophage-like cells (B and D). By day 7, density of cellular infiltrate surrounding the lesion cavity was also reduced with the NNZ-2566 treatment (F) compared to the vehicle treatment (E).

Upon gross microscopic evaluation, PBBI produced unilateral hemorrhagic lesions that predominately involve the frontal cortex and striatum (Figure 1A). NNZ-2566 treatment (Figure 1B) had no observable effect on reducing the size of the core lesion.

In the PBBI animals, inflammatory cell infiltration in tissue surrounding the primary lesion site was clearly observed with neutrophils and parenchymal monocytes/macrophages being the dominant cell types. NNZ-2566 suppressed PBBI induced inflammatory cell infiltration at 3 days following PBBI (Figure 1D) as compared to vehicle treatment (Figure 1C). By day 7, the majority of the inflammatory response was restricted to peri-lesional zones within the immediate region of the primary lesion, forming a wide band of reactive gliosis (Figure 1E) which was also decreased by NNZ-2566 treatment (Figure 1F).

### Microarray analysis of brain inflammatory gene expression responses to PBBI

**Figure 2:**
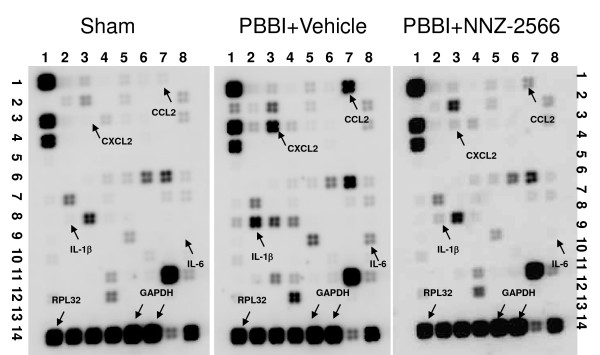
**Representative microarray images of inflammatory cytokine gene arrays from sham-PBBI, PBBI, and NNZ-2566 treated PBBI rats 4 hours after the injury are shown**. GAPDH and RPL32 served as internal controls. Genes for which robust changes in mRNA expression were observed are indicated by the arrows.

Microarray results performed with RNA isolated from the injured (right) brain hemisphere 4 h post-PBBI injury are shown in Figure 2. Expression of the internal controls, RPL32 and GAPDH genes, was similar in all groups. Conversely, PBBI produced robust increases in the expression of IL-1β, IL-6, Chemokine (C-C motif) ligand 2 (CCL2, also known as monocyte chemoattractant protein-1, MCP-1), and Chemokine (C-X-C motif) ligand 2 (CXCL2, also known as macrophage inflammatory protein 2-alpha, MIP2-α) genes that were attenuated by post-injury administration of NNZ-2566.

The effects of PBBI on the gene transcripts depicted in Figure 2 are summarized in Table 1. Following PBBI, 10 different chemokines and cytokines mRNAs showed a greater-than 2-fold up-regulation whereas only one cytokine mRNA was down-regulated greater than 2-fold. Treatment with NNZ-2566 robustly reduced the increase in transcription of the pro-inflammatory genes IL-1β, IL-6, CCL2, and CXCL2. Microarray results with RNA isolated from the contralateral (non-injured) brain hemisphere were the same as RNA results from sham brains.

**Table 1:** Oligo-DNA microarray analysis of cytokine genes that exhibited a ± 2.0 fold change in expression level between the left (uninjured) and right (injured) brain hemispheres (4 h post-injury).

**Gene Name**	**Gene Bank #**	**PBBI** **Regulation (fold change)**	**NNZ-2566/PBBI Regulation (fold change)**
Chemokine (C-C motif) ligand 2	NM_031530	↑ 21.2	↑ 4.0
Chemokine (C-X-C motif) ligand 2	NM_053647	↑ 20.0	↑ 4.6
Interleukin 6	NM_012589	↑ 12.4	↑ 5.1
Interleukin 1 beta	NM_031512	↑ 11.9	↑ 2.8
Interleukin 1 alpha	NM_017019	↑ 7.0	↑ 32
Interleukin 1 receptor, type II	NM_053953	↑ 6.8	↑ < 2 (1.1)
Chemokine (C-X-C motif) ligand 10	NM_139089	↑ 3.2	↑ 2.3
Chemokine (C-C motif) ligand 3	NM_013025	↑ 3.0	↑ 2.3
Interleukin 4 receptor	NM_133380	↑ 2.8	↑ < 2
Chemokine (C-C motif) ligand 20	NM_019233	↑ 2.7	↑ < 2 (1.0)
Interleukin 6 receptor	NM_017020	↑ < 2 (1.1)	↓ 2.4
CREB binding protein	NM_133381	↑ < 2 (1.1)	↓ 2.0
Lymphotoxin A	NM_080769	↓ 2.2	↓ < 2 (1.9)
Fibroblast growth factor receptor 3	NM_053429	↓ < 2 (1.5)	↓ 2.1
Transforming growth factor alpha	NM_012671	↓ < 2 (1.1)	↓ 2.2

The results of the microarray were quantified using GEArray Expression Analysis Suite. Data are pooled from 6 rats per group and expressed as folds over uninjured brain hemisphere.

### Inflammatory gene RNA expression profile with QRT-PCR

**Figure 3:**
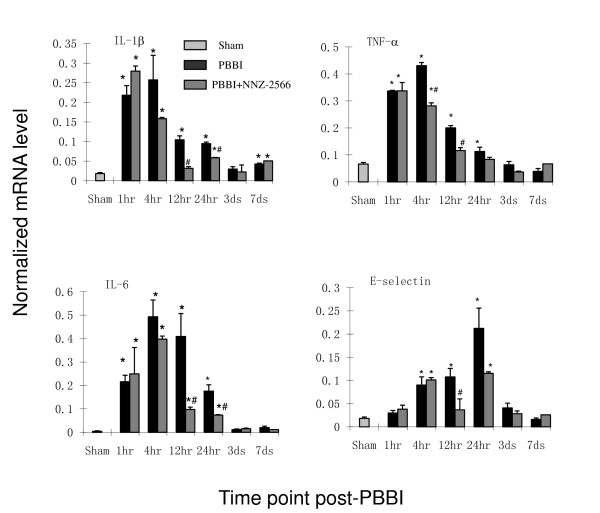
**Time-course of gene expression as measured by qRT-PCR**. The mRNA levels are given as the mean ± standard error (n = 6 per group) normalized to RPL32 levels for each sample. *P < 0.05 as compared to the sham and # *P *< 0.05 as compared to PBBI treated with the vehicle.

QRT-PCR was used to confirm and expand upon the results of the microarray screening by tracking the time course changes of the selected cytokines (IL-1β, IL-6, and TNF-α) as well as the cellular adhesion molecule (E-selectin) mRNA levels following PBBI (Figure 3; selected on the basis of our earlier findings using an ischemic brain injury model[27]). RNA expression from sham rats was consistently expressed with minimal change during various observation time points. TNF-α, IL-1β, and IL-6 were significantly elevated between 1 h and 24 h post-PBBI with peak expression levels occurring between 1 h and 4 h post-injury. Significant expression of E-selectin was slightly delayed (i.e. 4 h) and peaked at 24 h post-injury. Average peak increases in gene transcription for the vehicle-treated PBBI rats (compared to sham) were as follows: 60-fold for IL-6, 14-fold for E-selectin, 12-fold for IL-1β, and 6-fold for TNF-α. The increase in expression of inflammatory gene mRNAs subsided between 24 h to 3 days post-injury. NNZ-2566 treatment significantly reduced the elevation of IL-6 (79%), E-selectin (81%), IL-1β (76%) and TNF-α (72%) mRNA levels in the injured hemisphere at 12 h post-PBBI, with maximal inhibition occurring between 12 h and 24 h. The increase in expression of secondary chemokines like CCL2 and CXCL2 (> 10–15 folds) were also confirmed by QRT/PCR at 4 h post-injury (data are not shown). The effects of NNZ-2566 treatment on PBBI-induced up-regulation of these and other related secondary cytokines/chemokines are currently under evaluation.

### Cytokine levels quantified with ELISA array

**Figure 4:**
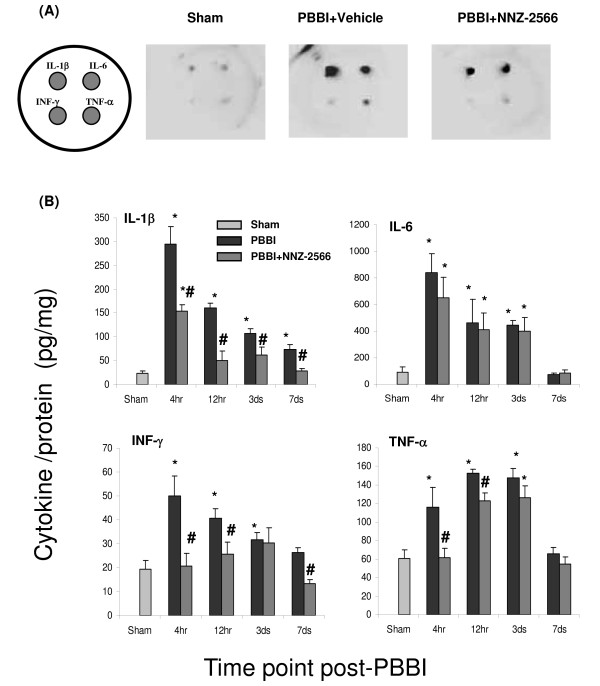
**Multiplex cytokine array analysis of cytokine levels following PBBI**. NNZ-2566 treatment is similar to that listed in legend of figure 3. (A) Schematic indicating the orientation of cytokines (IL-1β, IL6, INF-γ, and TNF-α) and representative images of chemiluminiscent intensity from the multiplex array (4 hours after PBBI). (B) Quantitative analysis from the multiplex array measurements. The cytokine levels are given as the mean ± standard error (n = 6 per group). *P < 0.05 as compared to the sham, and # *P *< 0.05 as compared to PBBI with the vehicle.

Levels of cytokines IL-1β, IL-6, interferon gamma (INF-γ) and TNF-α in brain tissue lysate at each time points measured using a commercially available ELISA array (that measured only these four cytokines simultaneously) are shown in Figure 4. The inflammatory cytokine levels in sham rats tissue from different time points were consistently presented in a low background. All four measured cytokine levels exhibited rapid increases at the early time points (4 h and 12 h post-injury) that, with the exception of IL-1β, subsided by day 7 post-injury. Further, whereas IL-1β and IL-6 showed a 15- to 19-fold increase in expression by 4 h post-PBBI, TNF-α and INF-γ exhibited only 2-to 3- fold increase. NNZ-2566 treatment did not affect the PBBI-induced up-regulation of IL-6 expression at any time point, but did produce significant reductions in the injury-induced up-regulation of IL-1β INF-γ, and TNF-α expression.

### Reactive gliosis

**Figure 5:**
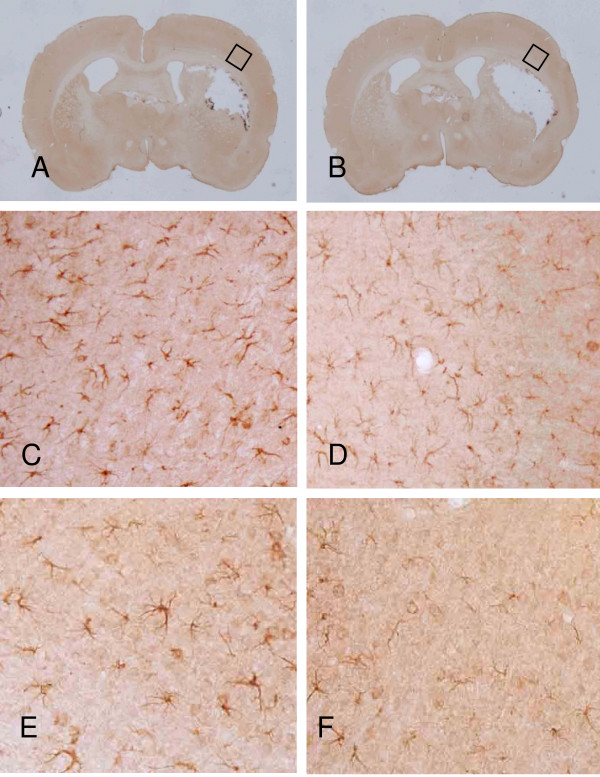
**PBBI-induced IL-1β immunoreactivity in vehicle (A) or NNZ-2566 (B) treated rats at 3 (C and D) or 7 days (E and F)**. IL-1β positive cells are visible in the region surrounding the lesion cavity at 3 days following PBBI (C) which are reduced following NNZ-2566 treatment (D). Panel E and F are representative photomicrographs of injured hemisphere at 7 days for PBBI (E) or PBBI + NNZ-2566 (F).

The changes in IL-1β immunoreactivity for both the vehicle and NNZ-2566 treated animals are illustrated in Figure 5. The peri-lesional region exhibited highly ramified IL-1β-positive microglia cells with a more abundant expression profile at 3 days post-PBBI and a less abundant expression at 7 days post-PBBI. NNZ-2566 treatment suppressed IL-1β expression in the injured brain hemisphere for up to 7 days post-PBBI.

## Discussion

Traumatic brain injury triggers an inflammatory response characterized by the invasion of circulating immune cells and release of pro-inflammatory cytokines [9,29]. In the present study, PBBI produced an acute, severe neuroinflammatory response that consisted of the release of pro-inflammatory cytokines, cellular adhesion molecules, neutrophil infiltration, and reactive microgliosis. However, post-injury administration of NNZ-2566 significantly attenuated PBBI-induced neuroinflammatory and neuropathological events.

Post-injury administration of NNZ-2566, using the optimal dose and treatment regimen selected from our most recent neuroprotection study [25], caused broad inhibition of mRNA in 10 of the associated pro-inflammatory chemokines and cytokines. Only one cytokine mRNA (lymphotoxin A) showed a marginal decrease in expression following PBBI, which was not affected by NNZ-2566. The current study focused on the inhibitory effect of NNZ-2566 on selected up-regulated cytokines detected by microarray and confirmed by qRT-PCR. The inhibitory effect of NNZ-2566 on these cytokine mRNA expressions appeared 4 h post-injury, much earlier than the infiltration of blood-derived leukocytes and activation of neural-glial cells, which in previous studies has been shown to occur as early as 6 hours following the brain injury [5-7].

Although our primary focus was pro-inflammatory cytokines, it is worth noting that we also observed robust upregulation of two secondary chemokines, CCL-2 and CXCL-2, in microarray screening and were verified by qRT-PCR at a single time point (4 h post-injury). However, evidence suggests that chemokine secretion by cells at inflammatory sites may occur in response to up-regulation of the major pro-inflammatory cytokines [70]. Follow-up studies are currently in progress to determine the effect of NNZ-2566 on this secondary pathway. Nevertheless, the main focus of this study was to determine the potential neuroprotective effects of NNZ-2566 on PBBI-induced up-regulation of major pro-inflammatory cytokines, such as IL-1β, TNF-α, and INF-γ.

These cytokines have been implicated in tissue damage *via *recruitment of inflammatory cells [30-33] and appear to play "dual roles" in CNS injury. Evidence suggests that these cytokines may exert deleterious effects during acute injury stages (4 h to 3 days) and a reparative effect, albeit at much lower expression levels, during more delayed recovery periods,[11,34,35]. In our studies, we have observed induction of these cytokines as early as 1 h to 4 h post injury, followed by a decline in their expression at later time points. Early induction could lead to the deleterious effects after PBBI and decline in cytokine expression over time may indicate lack of repair.

NNZ-2566 effectively suppressed IL-1β up-regulation caused by PBBI. The increased expression of IL-1β following PBBI was profound (i.e. 15 fold > sham levels) and prolonged (7 days) compared to moderate (2- to 3-fold) and short-lived (< 3 days post-injury) increases in TNF-α and INF-γ levels. The role of IL-1β in exacerbating neurodegeneration is well-documented and sustained post-injury up-regulation of IL-1β has been implicated in secondary neuronal loss [10,36,37]. NNZ-2566 significantly decreased (50–90%) the PBBI-induced surge in IL-1β mRNA and in protein levels quantified by qRT-PCR and ELISA cytokine array as early as 4 hours post-injury; an effect that was sustained out to 7 days. The ability of NNZ-2566 to exert prolonged suppression of injury-induced IL-1β expression may represent a critically important neuroprotective mechanism of this compound,

In various experimental settings TNF-α has been designated as a pro-inflammatory and harmful cytokine [35] causing deleterious effects that were attenuated by TNF inhibition [36]. Other studies, however, demonstrated that, while TNF-α may be detrimental during the acute brain injury [37], its expression is actually beneficial during chronic (> 24 h) post-injury periods [37-39] where it has been shown to play a reparative role in chronic inflammation and promote the accumulation of proliferating oligodendrocyte progenitors required for remyelination [39,40]. In the present study, PBBI resulted in significant up-regulation of TNF-α expression during the acute (1–24 h) post-injury phase (the time period when it is considered detrimental to neuronal cells) that slowly declined during the chronic (3–7 days) post-injury phase. Notably, NNZ-2566 treatment suppressed injury-induced TNF-α upregulation during the acute (4–12 h) post-injury time period, but had no prolonged effect on TNF-α expression. While we cannot rule out that this result may be linked to the duration (12 h) of NNZ-2566 administration, it may be pivotal in defining the neuroprotective benefits of NNZ-2566 and how it should be applied to achieve therapeutic benefits in brain injury therapy.

NNZ-2566 also protected against injury-induced up-regulation of INF-γ, a pro-inflammatory cytokine that orchestrates the trafficking of specific immune cells to inflammatory sites via up-regulation of adhesion molecules and chemokines, and promotes the activation of 'killer' cells and lysosome activity in macrophages [41]. In this study, NNZ-2566 reduced abnormal INF-γ levels during the acute injury stages (4 h and 12 h). However, this inhibitory effect was not observed at 3 days post-infusion.

In contrast to TNF-α, IL-1β, and IFN-γ, IL-6 was originally classified as a neuroprotective and anti-inflammatory cytokine with pleiotropic functions, and as a regulator of intracerebral homeostasis [42,43]. Its specific anti-inflammatory properties include the inhibition of TNF and induction of IL-1RA (interleukine 1 receptor antagonist) [38]. Its neuroprotective properties include the stimulation of NGF (nerve growth factor) production, defense against glutamate-mediated toxicity and oxidative stress, and promotion of revascularization [44]. This classification as a beneficial cytokine has been challenged by studies that demonstrate IL-6 contributes to adverse outcomes in autoimmune neuropathology [45] and promotes inflammation by influencing chemotaxis, which is correlated with up-regulated chemokine production and adhesion molecule expression [46,47]. However, most of the clinical data support the classification of IL-6 as a neuroreparative cytokine [35,42].

In our study, IL-6 mRNA up-regulation following injury was detected in the acute phase (within 24 hours) but returned to basal levels by 3 days post-injury. Although up-regulation of IL-6 mRNA was significantly reduced by NNZ-2566 treatment, elevation of IL-6 protein was not significantly altered. One plausible explanation for this discrepancy may be that treatment with NNZ-2566 shortens the stability of IL-6 mRNA, but may not have significant impact on IL-6 translational efficiency. This hypothesis is supported by evidence that IL-6 mRNA contains AU-rich elements (AREs) that are required to regulate the half-life of many cytokines mRNA [48,49].

We also analyzed the post-PBBI expression level of E-selectin, a tissue adhesion molecule that is induced by cytokines such as IL-1β and TNF-α [50,51]. E-selectin initiates low-affinity interaction between leukocytes and endothelial cells and promotes the recruitment and "rolling" of leukocytes along endothelial walls for accumulation and activation of leukocytes in the injured tissue [52-54]. We detected a strong up-regulation of E-selectin mRNA expression starting at 4 hours and peaking at 24 hours after PBBI in the injured hemisphere, trailing the upregulation of TNF and IL-1. This up-regulation of E-selectin transcript after PPBI may be associated with the recruitment of peripheral inflammatory cells, including neutrophils and macrophages, into the brain that may further aggravate the inflammatory response in the injured brain regions. Treatment with NNZ-2566 produced a significant inhibition of this adhesion molecule several hours following the inhibition of IL-1β and TNF-α RNA expression. These results suggest that NNZ-2566-mediated inhibition of PBBI-induced cytokine expression may, in turn, suppress the expression of E-selectin mRNA thus reducing leukocyte infiltration and subsequent neuroinflammation.

The concept of using anti-inflammatory or immunosuppressive compounds to treat brain injury is not new [13,55-59]. However, despite promising preclinical results these pharmacological strategies have failed to provide a benefit in clinical trials [60-62] suggesting that the complex processes of neuroinflammation cannot be efficiently interrupted by targeting just one single mediator of inflammation [60], or one single neuronal injury mechanism.

## Conclusion

This is the first study to report that NNZ-2566 effectively suppresses expression of multiple inflammatory mediators, IL-1β, TNF-α and IFN-γ and E-selectin, and inhibits both acute and delayed neuroinflammation following PBBI. These results combined with those of our recent studies demonstrating promotion of functional recovery and attenuation of PBBI-induced inflammation and apoptosis by NNZ-2566 [25] provide further support for its use as a therapeutic agent for brain injury.

## List of abbreviations

AREs: AU-rich elements; CCL2: chemokine (C-C motif) ligand 2; CXCL2: chemokine (C-X-C motif) ligand 2; cRNA: complementary RNA; GAPDH: glyceraldehyde-3-phosphate dehydrogenase; GPE: Glycine-Proline-Glutamate (Glypromate ^®^; Neuren Pharmaceuticals); IGF-1: insulin-like growth factor-1; IL-1β interleukin 1 beta; IL-1RA: interleukine 1 receptor antagonist; IL-6: interleukin 6; INF-γ: interferon gamma; mRNA: messenger RNA; NGF: nerve growth factor; PBBI: penetrating ballistic-like brain injury; qRT-PCR: quantitative reverse transcriptase polymerase chain reaction; RPL32: Ribosomal protein L32; TBI: traumatic brain injury; TNF-α: tumor necrosis factor alpha.

## Competing interests

The authors declare that they have no competing interests.

## Authors' contributions

HHW designed and conducted the study, analyzed data, prepared the figures and wrote the manuscript, XCML, FCT and JRD participated in study design and preparation of the manuscript. DAS, AW and CY participated in manuscript preparation. AW and HHW performed the QRT-PCR experiments. All authors have read and approved the final manuscript.

## References

[B1] D'Ambrosio R, Perucca E (2004). Epilepsy after head injury. Curr Opin Neurol.

[B2] Maas AI, Stocchetti N, Bullock R (2008). Moderate and severe traumatic brain injury in adults. Lancet Neurol.

[B3] Langlois JAR-BW, Thomas KE (2006). Traumatic Brain Injury in the United States: Emergency Department Visits, Hospitalizations, and Deaths.

[B4] Thurman DJAC, Browne D, Dunn KA, Guerroro J, Johnson R, Johnson V, Langlois J, Pilkey D, Sniezek JE, Toal S (1999). Traumatic Brain Injury in the United States: A Report to Congress.

[B5] Williams AJ, Hartings JA, Lu XC, Rolli ML, Dave JR, Tortella FC (2005). Characterization of a new rat model of penetrating ballistic brain injury. J Neurotrauma.

[B6] Williams AJ, Hartings JA, Lu XC, Rolli ML, Tortella FC (2006). Penetrating ballistic-like brain injury in the rat: differential time courses of hemorrhage, cell death, inflammation, and remote degeneration. J Neurotrauma.

[B7] Williams AJ, Wei HH, Dave JR, Tortella FC (2007). Acute and delayed neuroinflammatory response following experimental penetrating ballistic brain injury in the rat. J Neuroinflammation.

[B8] Werner C, Engelhard K (2007). Pathophysiology of traumatic brain injury. Br J Anaesth.

[B9] Lucas SM, Rothwell NJ, Gibson RM (2006). The role of inflammation in CNS injury and disease. Br J Pharmacol.

[B10] Simi A, Tsakiri N, Wang P, Rothwell NJ (2007). Interleukin-1 and inflammatory neurodegeneration. Biochem Soc Trans.

[B11] Wang CX, Shuaib A (2002). Involvement of inflammatory cytokines in central nervous system injury. Prog Neurobiol.

[B12] Williams AJ, Dave JR, Tortella FC (2006). Neuroprotection with the proteasome inhibitor MLN519 in focal ischemic brain injury: relation to nuclear factor kappaB (NF-kappaB), inflammatory gene expression, and leukocyte infiltration. Neurochem Int.

[B13] Bye N, Habgood MD, Callaway JK, Malakooti N, Potter A, Kossmann T, Morganti-Kossmann MC (2007). Transient neuroprotection by minocycline following traumatic brain injury is associated with attenuated microglial activation but no changes in cell apoptosis or neutrophil infiltration. Exp Neurol.

[B14] Sara VR, Carlsson-Skwirut C, Bergman T, Jornvall H, Roberts PJ, Crawford M, Hakansson LN, Civalero I, Nordberg A (1989). Identification of Gly-Pro-Glu (GPE), the aminoterminal tripeptide of insulin-like growth factor 1 which is truncated in brain, as a novel neuroactive peptide. Biochem Biophys Res Commun.

[B15] Sara VR, Carlsson-Skwirut C, Drakenberg K, Giacobini MB, Hakansson L, Mirmiran M, Nordberg A, Olson L, Reinecke M, Stahlbom PA (1993). The biological role of truncated insulin-like growth factor-1 and the tripeptide GPE in the central nervous system. Ann N Y Acad Sci.

[B16] Yamamoto H, Murphy LJ (1994). Generation of des-(1–3) insulin-like growth factor-I in serum by an acid protease. Endocrinology.

[B17] Yamamoto H, Murphy LJ (1995). Enzymatic conversion of IGF-I to des(1–3)IGF-I in rat serum and tissues: a further potential site of growth hormone regulation of IGF-I action. J Endocrinol.

[B18] Bourguignon J, Gerard A (1999). Role of insulin-like growth factor binding proteins in limitation of IGF-I degradation into the N-methyl-D-aspartate receptor antagonist GPE: evidence from gonadotrophin-releasing hormone secretion in vitro at two developmental stages. Brain Res.

[B19] Carlsson-Skwirut C, Lake M, Hartmanis M, Hall K, Sara VR (1989). A comparison of the biological activity of the recombinant intact and truncated insulin-like growth factor 1 (IGF-1). Biochim Biophys Acta.

[B20] Alexi T, Hughes PE, van Roon-Mom WM, Faull RL, Williams CE, Clark RG, Gluckman PD (1999). The IGF-I amino-terminal tripeptide glycine-proline-glutamate (GPE) is neuroprotective to striatum in the quinolinic acid lesion animal model of Huntington's disease. Exp Neurol.

[B21] Saura J, Curatolo L, Williams CE, Gatti S, Benatti L, Peeters C, Guan J, Dragunow M, Post C, Faull RL (1999). Neuroprotective effects of Gly-Pro-Glu, the N-terminal tripeptide of IGF-1, in the hippocampus in vitro. Neuroreport.

[B22] Batchelor DC, Lin H, Wen JY, Keven C, Van Zijl PL, Breier BH, Gluckman PD, Thomas GB (2003). Pharmacokinetics of glycine-proline-glutamate, the N-terminal tripeptide of insulin-like growth factor-1, in rats. Anal Biochem.

[B23] Guan J, Thomas GB, Lin H, Mathai S, Bachelor DC, George S, Gluckman PD (2004). Neuroprotective effects of the N-terminal tripeptide of insulin-like growth factor-1, glycine-proline-glutamate (GPE) following intravenous infusion in hypoxic-ischemic adult rats. Neuropharmacology.

[B24] Guan J (2008). Insulin-like growth factor-1 and its derivatives: potential pharmaceutical application for ischemic brain injury. Recent Patents CNS Drug Discov.

[B25] Lu XC, Chen RW, Yao C, Wei H, Yang X, Liao Z, Dave JR, Tortella FC (2566). NNZ- a glypromate analog, improves functional recovery and attenuates apoptosis and inflammation in a rat model of penetrating ballistic-type brain injury. J Neurotrauma.

[B26] Williams AJ, Ling GS, Tortella FC (2006). Severity level and injury track determine outcome following a penetrating ballistic-like brain injury in the rat. Neurosci Lett.

[B27] Clark RK, Lee EV, Fish CJ, White RF, Price WJ, Jonak ZL, Feuerstein GZ, Barone FC (1993). Development of tissue damage, inflammation and resolution following stroke: an immunohistochemical and quantitative planimetric study. Brain Res Bull.

[B28] Berti R, Williams AJ, Moffett JR, Hale SL, Velarde LC, Elliott PJ, Yao C, Dave JR, Tortella FC (2002). Quantitative real-time RT-PCR analysis of inflammatory gene expression associated with ischemia-reperfusion brain injury. J Cereb Blood Flow Metab.

[B29] Ghirnikar RS, Lee YL, Eng LF (1998). Inflammation in traumatic brain injury: role of cytokines and chemokines. Neurochem Res.

[B30] Bian ZM, Elner SG, Yoshida A, Kunkel SL, Su J, Elner VM (2001). Activation of p38, ERK1/2 and NIK pathways is required for IL-1beta and TNF-alpha-induced chemokine expression in human retinal pigment epithelial cells. Exp Eye Res.

[B31] Elner SG, Strieter RM, Elner VM, Rollins BJ, Del Monte MA, Kunkel SL (1991). Monocyte chemotactic protein gene expression by cytokine-treated human retinal pigment epithelial cells. Lab Invest.

[B32] Elner VM, Strieter RM, Elner SG, Baggiolini M, Lindley I, Kunkel SL (1990). Neutrophil chemotactic factor (IL-8) gene expression by cytokine-treated retinal pigment epithelial cells. Am J Pathol.

[B33] Amantea D, Nappi G, Bernardi G, Bagetta G, Corasaniti MT (2009). Post-ischemic brain damage: pathophysiology and role of inflammatory mediators. FEBS J.

[B34] Correale J, Villa A (2004). The neuroprotective role of inflammation in nervous system injuries. J Neurol.

[B35] Schmidt OI, Heyde CE, Ertel W, Stahel PF (2005). Closed head injury – an inflammatory disease?. Brain Res Brain Res Rev.

[B36] Lavine SD, Hofman FM, Zlokovic BV (1998). Circulating antibody against tumor necrosis factor-alpha protects rat brain from reperfusion injury. J Cereb Blood Flow Metab.

[B37] Scherbel U, Raghupathi R, Nakamura M, Saatman KE, Trojanowski JQ, Neugebauer E, Marino MW, McIntosh TK (1999). Differential acute and chronic responses of tumor necrosis factor-deficient mice to experimental brain injury. Proc Natl Acad Sci USA.

[B38] Stahel PF, Shohami E, Younis FM, Kariya K, Otto VI, Lenzlinger PM, Grosjean MB, Eugster HP, Trentz O, Kossmann T, Morganti-Kossmann MC (2000). Experimental closed head injury: analysis of neurological outcome, blood-brain barrier dysfunction, intracranial neutrophil infiltration, and neuronal cell death in mice deficient in genes for pro-inflammatory cytokines. J Cereb Blood Flow Metab.

[B39] Shohami E, Ginis I, Hallenbeck JM (1999). Dual role of tumor necrosis factor alpha in brain injury. Cytokine Growth Factor Rev.

[B40] Arnett HA, Mason J, Marino M, Suzuki K, Matsushima GK, Ting JP (2001). TNF alpha promotes proliferation of oligodendrocyte progenitors and remyelination. Nat Neurosci.

[B41] Schroder K, Hertzog PJ, Ravasi T, Hume DA (2004). Interferon-gamma: an overview of signals, mechanisms and functions. J Leukoc Biol.

[B42] Gruol DL, Nelson TE (1997). Physiological and pathological roles of interleukin-6 in the central nervous system. Mol Neurobiol.

[B43] Opal SM, DePalo VA (2000). Anti-inflammatory cytokines. Chest.

[B44] Morganti-Kossmann MC, Satgunaseelan L, Bye N, Kossmann T (2007). Modulation of immune response by head injury. Injury.

[B45] Eugster HP, Frei K, Kopf M, Lassmann H, Fontana A (1998). IL-6-deficient mice resist myelin oligodendrocyte glycoprotein-induced autoimmune encephalomyelitis. Eur J Immunol.

[B46] Romano M, Sironi M, Toniatti C, Polentarutti N, Fruscella P, Ghezzi P, Faggioni R, Luini W, van Hinsbergh V, Sozzani S (1997). Role of IL-6 and its soluble receptor in induction of chemokines and leukocyte recruitment. Immunity.

[B47] Modur V, Li Y, Zimmerman GA, Prescott SM, McIntyre TM (1997). Retrograde inflammatory signaling from neutrophils to endothelial cells by soluble interleukin-6 receptor alpha. J Clin Invest.

[B48] Asirvatham AJ, Magner WJ, Tomasi TB (2009). miRNA regulation of cytokine genes. Cytokine.

[B49] Ross J (1995). mRNA stability in mammalian cells. Microbiol Rev.

[B50] Haraldsen G, Kvale D, Lien B, Farstad IN, Brandtzaeg P (1996). Cytokine-regulated expression of E-selectin, intercellular adhesion molecule-1 (ICAM-1), and vascular cell adhesion molecule-1 (VCAM-1) in human microvascular endothelial cells. J Immunol.

[B51] Ray KP, Farrow S, Daly M, Talabot F, Searle N (1997). Induction of the E-selectin promoter by interleukin 1 and tumour necrosis factor alpha, and inhibition by glucocorticoids. Biochem J.

[B52] Clark RS, Carlos TM, Schiding JK, Bree M, Fireman LA, DeKosky ST, Kochanek PM (1996). Antibodies against Mac-1 attenuate neutrophil accumulation after traumatic brain injury in rats. J Neurotrauma.

[B53] Berti R, Williams AJ, Velarde LC, Moffett JR, Elliott PJ, Adams J, Yao C, Dave JR, Tortella FC (2003). Effect of the proteasome inhibitor MLN519 on the expression of inflammatory molecules following middle cerebral artery occlusion and reperfusion in the rat. Neurotox Res.

[B54] Ley K, Laudanna C, Cybulsky MI, Nourshargh S (2007). Getting to the site of inflammation: the leukocyte adhesion cascade updated. Nat Rev Immunol.

[B55] Balasingam V, Yong VW (1996). Attenuation of astroglial reactivity by interleukin-10. J Neurosci.

[B56] Knoblach SM, Faden AI (1998). Interleukin-10 improves outcome and alters proinflammatory cytokine expression after experimental traumatic brain injury. Exp Neurol.

[B57] Relton JK, Rothwell NJ (1992). Interleukin-1 receptor antagonist inhibits ischaemic and excitotoxic neuronal damage in the rat. Brain Res Bull.

[B58] Koprich JB, Reske-Nielsen C, Mithal P, Isacson O (2008). Neuroinflammation mediated by IL-1beta increases susceptibility of dopamine neurons to degeneration in an animal model of Parkinson's disease. J Neuroinflammation.

[B59] Sanchez Mejia RO, Ona VO, Li M, Friedlander RM (2001). Minocycline reduces traumatic brain injury-mediated caspase-1 activation, tissue damage, and neurological dysfunction. Neurosurgery.

[B60] Bullock MR, Lyeth BG, Muizelaar JP (1999). Current status of neuroprotection trials for traumatic brain injury: lessons from animal models and clinical studies. Neurosurgery.

[B61] Narayan RK, Michel ME, Ansell B, Baethmann A, Biegon A, Bracken MB, Bullock MR, Choi SC, Clifton GL, Contant CF (2002). Clinical trials in head injury. J Neurotrauma.

[B62] Bayir H, Clark RS, Kochanek PM (2003). Promising strategies to minimize secondary brain injury after head trauma. Crit Care Med.

